# Prognosis of Adrenal Oncocytic Neoplasms (AONs): Literature Review of 287 Cases and Presentation of the Oldest Patient

**DOI:** 10.3390/jcm12216925

**Published:** 2023-11-04

**Authors:** Enrico Coppola Bottazzi, Claudio Gambardella, Federico Maria Mongardini, Serafino Vanella, Adele Noviello, Tommaso Palma, Rosa Murano, Giovanni De Chiara, Giovanni Conzo, Ludovico Docimo, Francesco Crafa

**Affiliations:** 1 Oncological and General Surgery Unit, “St. Giuseppe Moscati” Hospital of National Relevance and High Specialty, 83100 Avellino, Italy; enrico.coppolabottazzi@aornmoscati.it (E.C.B.); nekroma@yahoo.it (S.V.); adele.noviello@aornmoscati.it (A.N.); rosa.murano@aornmoscati.it (R.M.);; 2 Division of General, Oncological, Mini-Invasive and Obesity Surgery, University of Study of Campania “Luigi Vanvitelli”, 80138 Naples, Italy; f.mongardini@gmail.com (F.M.M.); giovanni.conzo@unicampania.it (G.C.);; 3 Pathological Anatomy and Histology Unit, “St. Giuseppe Moscati” Hospital of National Relevance and High Specialty, 83100 Avellino, Italy; giovanni.dechiara@aornmoscati.it

**Keywords:** adrenal oncocytic neoplasms, adrenal oncocytoma, oncocytic neoplasm, adrenal oncocytic carcinoma

## Abstract

Introduction: The adrenocortical oncocytic neoplasms (AONs) are rare tumors of the adrenal gland, classified as oncocytoma (AO), oncocytic neoplasm of uncertain malignant potential (AONUMP) and oncocytic carcinoma (AOC). The aim of this study was to perform a review of the literature, in order to evaluate the prognosis of these rare cancers. We also reported the oldest patient with AON. Methods: A comprehensive literature review using as key words “adrenal oncocytoma”, “adrenal oncocytic neoplasm”, and “adrenal oncocytic carcinoma” was performed. Report of the case: We report the case of an 88-year-old woman receiving a left open adrenalectomy for an AON (15 × 10 × 8 cm). The considerable size and weight together with the presence of necrosis were indicative for a lesion with an uncertain potential for malignancy, according to Weiss modified criteria. After two years, the patient was free from any sign of recurrence. Results: Only 287 AONs were detected in the scientific literature, exploring OVID, MEDLINE, PubMed and SCOPUS as dataset. These tumors are usually incidentalomas with an unpredictable malignant potential. Surgical resection remains the mainstay of treatment for AON. Conclusion: AO and AONUMP have an excellent prognosis and a low mortality rate, with only three cases of recurrence reported in the literature and one metastatic case four years after first adrenal surgery. In contrast, AOC carries a high risk of local relapses, distant metastasis, and a significantly higher mortality rate (30%). Surgical resection remains the primary treatment for adrenal oncocytic neoplasms.

## 1. Introduction

Oncocytes are large polygonal or round cells with abundant granular and eosinophilic cytoplasm that are demonstrably packed with mitochondria on ultrastructural examination. Foci of oncocytic change are not uncommon in conventional adrenocortical neoplasms. Hamperl first used the term oncocytosis in 1931 [1]. These oncocytic cells were defined in salivary gland tumors and were added to the American literature by Jaffe [2,3].

Oncocytic neoplasms (ONs) are, by definition, composed exclusively or almost exclusively of oncocytes [4]. ONs can occur in various organs, notably the kidney, thyroid, pituitary, salivary, parathyroid, and lacrimal glands as well as the skin and respiratory and gastrointestinal tracts; the adrenal gland is a very uncommon location for ONs. 

Kakimoto S et al. described in 1986 the first case of adrenocortical oncocytic neoplasms (AONs), as a rare tumor of the adrenal gland [5]. It is important to differentiate them from pheochromocytomas, adrenocortical carcinoma and metastasis. These tumors are usually incidentalomas, unwittingly detected during radiological investigations performed for other medical reasons. They are usually benign lesions with an unpredictable malignant potential and mostly not secretory.

There is no single parameter to discriminate between benign and malignant AONs, and they are classified regarding their biological behavior by a combination of histological features according to the Lin–Weiss–Bisceglia system (LWB) [6].

AONs are classified, regarding their malignant potential, as oncocytoma (AO), oncocytic neoplasm of uncertain malignant potential (AONUMP), and oncocytic carcinoma (AOC) [7].

The aim of this study was to perform a review of the literature, in order to evaluate the prognosis of these rare cancers. Furthermore, we present the case of adrenal oncocytoma in the oldest patient in the literature.

## 2. Materials and Methods

Using the PubMed database, a systematic review of the current literature was carried out, up to May 2023. The Medical Subject Headings (MeSH) search terms used were oncocytic neoplasms, adrenocortical oncocytic neoplasms, adrenal tumors, adrenocortical oncocytomas. The authors observed that the adrenal AONs were rare. The keywords “oncocytic neoplasms”, “adrenocortical oncocytomas” and “adrenocortical oncocytic neoplasms” were used for the research. Several combinations of the keywords were utilized. The last research was concluded in May 2023. No language restrictions were imposed. The final article was realized in accordance with the Preferred Reporting Items for Systematic Reviews and Meta-Analyses (PRISMA) guidelines [8]. Moreover, the eligible articles were selected according to the modified Newcastle–Ottawa scale in order to satisfy the requirements of the current review. The scale range is from 0 to 9. The studies included were those presenting a score of 6 or higher [9]. The provided data, taken from the included studies, consisted of the first author’s name, the year of data collection and publication, the country of origin, characteristics of the study population, and the number of adrenocortical oncocytic neoplasm patients. Data on age, sex, location, size and weight, hormonal hyperfunction, Lin–Weiss–Bisceglia system follow-up and mortality were collected.

The study’s inclusion criteria involved patients with a confirmed histopathological diagnosis of adrenocortical oncocytic neoplasm.

A total of 138 articles were identified (118 full-text publications and 20 abstracts containing a complete description). Fifteen were case series, and all others single clinical cases; two abstracts were excluded due to the absence of information.

## 3. Case Presentation

**Figure 1:**
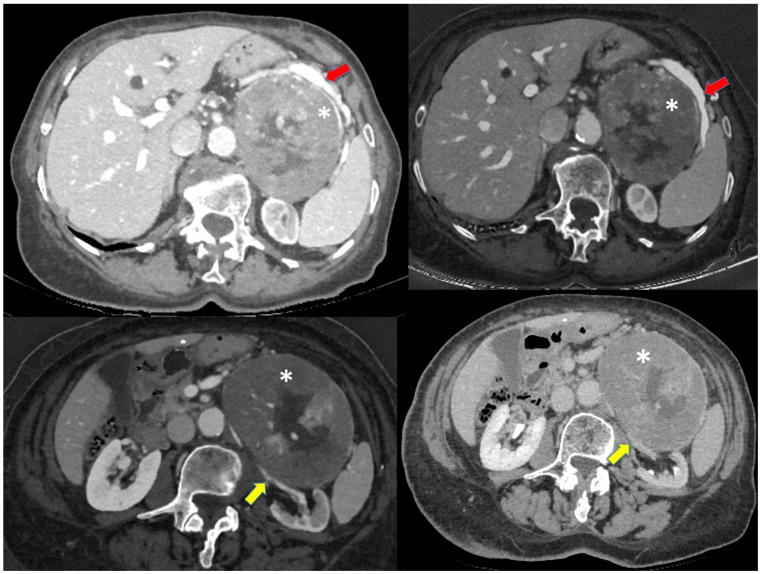
CT-scan image: left adrenal mass capsulated and not homogeneous, displacing the pancreatic body and the splenic vessels anteriorly and the kidney and renal vessels inferiorly. The red arrow indicates the splenic artery displaced by the mass, the yellow arrow indicates the left renal vessels, the asterisk indicates the adrenal mass.

An 88-year-old woman, diagnosed with pulmonary hypertension and experiencing difficulty walking following hip replacement surgery, underwent an abdominal ultrasound due to constipation and abdominal swelling, in the absence of other symptoms. The ultrasound revealed evidence of a small adrenal mass measuring less than 5 cm. A subsequent CT scan (Figure 1) performed a year later showed a large, solid mass (15 × 10 × 8 cm) encapsulated and not homogeneous, located in the left adrenal region. This mass displaced the pancreatic body and splenic vessels anteriorly, as well as the kidney and renal vessels inferiorly. Given the patient’s age, no further instrumental investigations such as MRI or PET scans were conducted.

Endocrine function tests did not show any abnormalities, confirming the suspicion of a non-functioning adrenal mass. Due to the tumor’s significant size and rapid growth, a left open adrenalectomy, with complete excision of the mass and preservation of the spleen and kidney, was performed. The postoperative course proceeded without complications; the patient was mobilized on the first day and discharged on the fifth. After two years of follow-up, she was disease-free.

**Figure 2:**
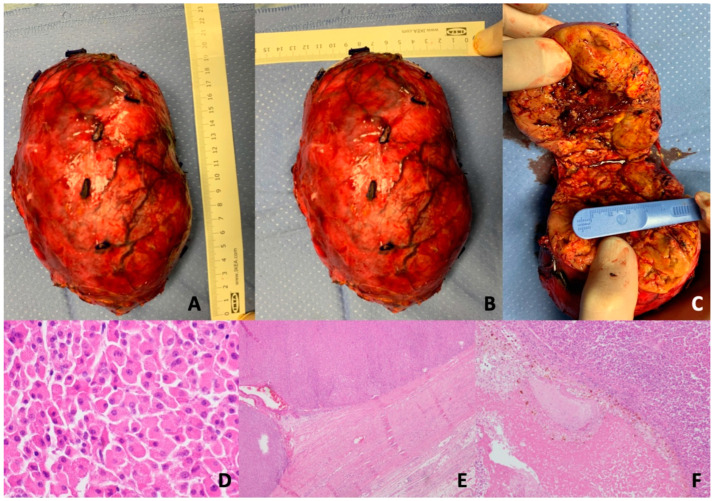
Macroscopically: (**A**,**B**) adrenal tumor mass (17 × 13 cm); (**C**) cut surface of the mass, homogeneous, well encapsulated and with central area of necrosis. Microscopically: (**D**) oncocytoma with abundant granular eosinophilic cytoplasm (magnification 60×); (**E**) an intact capsule (magnification 4×); (**F**) necrosis areas (magnification 20×).

Macroscopic examination revealed a rounded, smooth mass measuring 17 × 13 × 10 cm and weighing 730 g. The cut surface exhibited lobulated areas with yellowish and brownish regions, enclosed by a thin fibrous capsule (Figure 2).

Microscopic findings indicated a well-circumscribed, partly encapsulated tumor composed of nests and trabeculae of large polygonal cells with abundant eosinophilic granular cytoplasm. No venous invasion, high mitotic rate, or atypical mitoses were observed. While there were focal areas of necrosis, there was no evidence of capsular or sinusoidal invasion (Figure 2).

**Figure 3:**
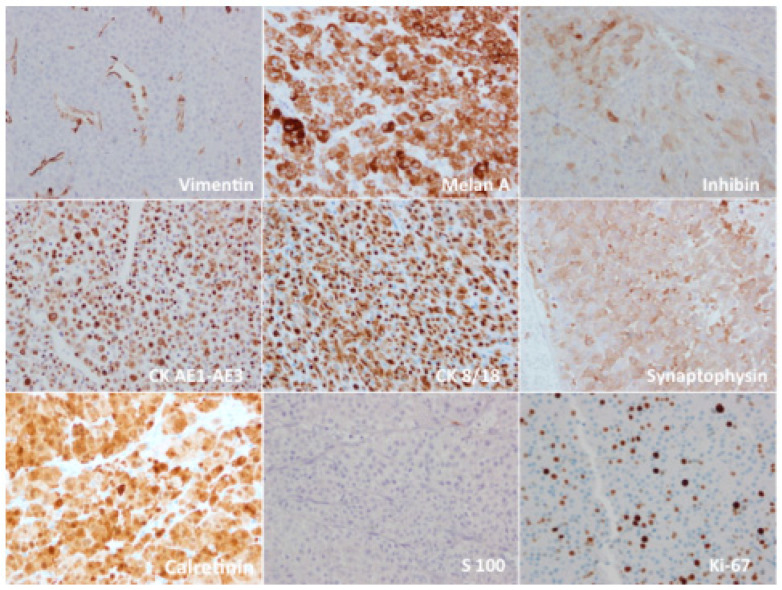
Immunohistochemistry study revealed: positivity for melan-A, inhibin, cytokeratins AE1-AE3, cytokeratin 8/18, synaptophysin, and calretinin; negativity for vimentin and S100 and Ki67/MIB1 proliferative activity of 20%. (Magnification 40×).

Immunohistochemical studies (Figure 3) revealed that the tumor cells were diffusely and strongly positive for melan-A, cytokeratins AE1-AE3, cytokeratin 8/18, synaptophysin, and calretinin, and focally and weakly positive for inhibin. Staining for Ki67/MIB1 showed a proliferative activity of 20%. Notably, the tumor cells did not react with vimentin, S100, p53, chromogranin, e-cadherin, CD 117, CD 10, PAX 8, or epithelial membrane antigen (Figure 3).

The morphological features were typical of an AON. The substantial size and weight of the tumor, along with the presence of necrotic areas, were factors considered indicative of a lesion with uncertain potential for malignancy, in accordance with the Weiss modified criteria [10].

Currently, two years after the resection of the adrenal mass, there are no signs of recurrence.

## 4. Results

Data of 136 articles were included in the review, comprising 287 cases of AONs, including our case. Demographic data of patients with AONs are shown in Table 1. AON patients presented a mean age at diagnosis of 44.2 years (range 3.5–87 years) and a female-to-male ratio of 1.9/1. AONs occurred with a slight prevalence on the left side (L/R: 1.4/1); in three cases (1.2%), they were bilateral [11,12,13], and in nine, the origin was ectopic [3,14,15,16,17,18,19,20,21].

The masses had an average diameter of 8.9 cm (range 2–28 cm) and an average weight of 467 g (range 8–5720 g) [22,23]. They were usually non-functional in 68% of cases, with a non-functional/functional ratio of 2.1/1.

As shown in Table 2, hyperandrogenism and Cushing syndrome were present in 13.2% and 8% of the AON patients, respectively; in 2.9% of cases, hypercortisolism and hyperandrogenism were simultaneous manifestations of adrenal oncocytoma. Functioning forms such as mimicking pheochromocytoma [24,25,26,27,28], Conn syndrome [12] and inflammatory IL-6 secretion [29,30] were rarely described in the literature.

Moreover, of the 278 cases in which the Lin–Weiss–Bisceglia system was applicable, (Table 1), 42% were diagnosed as oncocytomas (AOs), 28% as oncocytic neoplasms of uncertain malignant potential (AONUMPs) and 30% as oncocytic carcinomas (AOCs).

The follow-up (Table 3), reported in 135 cases (47.1%), was on average of 41.3 months for AO, 34.8 for AONUMP and 32.5 for AOC. Only two cases of recurrence in the AO group were reported [22,31]; only one case of local recurrence and hepatic metastasis appearing 4 years after first adrenal surgery was detected in the AONUMP group [32]. Conversely, seven cases of local recurrence and five of distant metastases (hepatic, bone and pulmonary) were reported in the malignant AOC group.

Percentages were calculated for those 135 cases with available follow-up information.

In the followed-up cases, mortality was absent in the AO group; only two deaths 4.2%) were reported in the AONUMP group, and nine deaths (30%) in the AOC group.

Table 4 reports the characteristics of death in the population with oncocytic adrenal neoplasm; from the 12 patients died with a diagnosis of adrenal oncocytoma, one was excluded because of synchronous metastatic rhabdomyosarcoma [23].

The average age was 48 years, with eight females and three males. Eight cases involved the left adrenal gland, while only three were located on the right one. The average size and weight of the tumors were 14.5 cm and 1067 g, respectively. Only two were functional neoplasms, associated with conditions such as hyperandrogenism or Cushing syndrome. According to LWB criteria, two were oncocytic neoplasms of uncertain malignant potential, and the other nine were carcinomas. The average follow-up of these patients was 2 years; only in one case, death was reported within 1 month of surgery.

## 5. Discussion

Adrenocortical oncocytic neoplasms (AONs) are exceptionally rare tumors. Differently from the previously described analysis—where only about 160 cases have been reported in the literature—we have identified 287 AON cases [36]. AONs were located in the adrenal cortex, with only one case reported by Chisté M. et al. within the adrenal medulla [37]. These tumors were more frequent in females (1.9/1), two of which were pregnant [38,39], and they may occur in all age groups, with a predominant occurrence approximately in the fourth and fifth decades of life (mean age 44.2 years); Subbiah S. et al. described a case of adrenocortical oncocytoma in the youngest patient, a girl aged 3-and-a-half years with heterosexual precocity [40].

In terms of location, AONs were found to have a slight prevalence on the left side (L/R: 1.4/1), which was lower than previously reported in the literature (L/R 3.5/1) [7]. In nine cases, the origin was ectopic, with four occurring in the retroperitoneal region [16,17,19,20,21], and the others located at the posteroinferior region of the cecum [3], hepatic [14], renal [15], and lumbar spine [18].

Most oncocytic tumors arising from endocrine organs were nonfunctional, as in our case, and often asymptomatic. In agreement with literature reports, about 30% were functional (32%). Hyperandrogenism and Cushing syndromes were the conditions most frequently associated with AONs; the simultaneous presence of virilization and subclinical Cushing was described in three of the functioning tumors [31,41,42].

Goel T et al. in 2007 first described a case of a 48-year-old male with oncocytic left adrenal mass who presented with hypertension and high urinary catecholamines; they concluded that functional adrenal oncocytoma mimicking pheochromocytoma should always be considered a possibility [24]. Subsequently, four other, similar cases of adrenal oncocytoma mimicking pheochromocytoma were reported in the literature [25,26,27,28]: tumors with clinical and laboratory findings consistent with pheochromocytoma that, however, had histopathologic features of an adrenal cortical neoplasm with predominant features of oncocytoma.

Peynirci H. et al. described two cases of primary hyperaldosteronism (Conn syndrome) among functional oncocytomas (0.7%) [12]; in contrast, Terui K. et al. reported the case of a non-functioning oncocytoma of 5.4 × 3.7 cm associated with an aldosterone-producing adenoma located around a margin of the oncocytoma that had an elongated configuration with a cross-sectional size of 3.5 × 0.5 cm [43].

Wong et al., in their series, described a case of a male with gynecomastia associated with elevated serum prolactin and estradiol [22]. Moreover, in the literature, two cases were reported of hypertension among non-secreting tumors that resolved after removal of the adrenal oncocytoma [33,44].

In 2017, the World Health Organization first recognized and classified the oncocytic variant as a distinctive subtype of ACCs [45]. In our review, 30% of the masses were malignant. The Weiss system has been adopted as the standard for the assessment and categorization of adrenocortical neoplasms [46]. Nevertheless, due to the paucity of reported cases of oncocytic neoplasms of the adrenal gland and the scarce follow-up data, the Lin–Weiss–Bisceglia system was adopted to revise the former criteria and aid the diagnosis of this unique tumor [6]. The major criteria include mitotic rate (more than five mitotic figures per 50 high-power fields), with atypical mitoses and venous invasion. The minor criteria include large tumor (>10 cm and/or >200 g), necrosis, and capsular and sinusoidal invasion. The presence of any major criterion would be diagnosed as malignant, and the presence of any minor criterion would be diagnosed as borderline or uncertain malignant potential, while the absence of any major or minor criteria would be diagnosed as benign [31].

Juliano JJ et al. reported a case of recurrent metastatic adrenal oncocytoma with the longest follow-up of about 15 years [47]. Metastatic spread was not previously reported in the scientific literature about adrenal cortical oncocytomas. Given the sites of metastases (bone, liver, lung), it seems to have a propensity for hematogenous spread. We can confirm that, to the best of our knowledge, there have been no published reports of lymph node metastases associated with these cases.

As demonstrated by Renaudin K. et al., malignant oncocytic adrenocortical tumors appeared to have a better prognosis than conventional adrenocortical carcinomas, but the corresponding hazard ratio did not reach statistical significance (hazard ratio = 0.45, confidence interval (0.13–1.59)) [48]; similar trends were observed even after excluding patients who were not treated with mitotane.

The imaging characterization of adrenal tumors relies on CT and MRI criteria, which include tumor size, morphological features, pre- and post-contrast CT densitometry, as well as MRI pre- and post-contrast signal intensity characteristics. When CT and/or MRI images present uncertainty in tumor characterization, functional radionuclide modalities such as 2-(18F) FDG PET/CT or PET-MRI hybrid imaging may be employed. It is important to note that imaging findings alone, even with the addition of more advanced imaging techniques, may not be sufficient to characterize adrenal oncocytomas. It is recommended to use integrated morpho-functional imaging to characterize adrenal tumors. However, since adrenal oncocytomas can sometimes exhibit 2-(18F) FDG uptake, there is a risk of overestimating malignancy. As FDG-avid masses, adrenal oncocytomas are known to be causes of false-positive PET results [19,45,49,50,51]. The diagnosis of adrenocortical oncocytoma mainly depends on the pathological examination. Fine-needle aspiration cytology appears to be a useful tool for confirming the diagnosis preoperatively. However, due to the substantial size of the tumor and the potential presence of heterogeneous areas, this technique may not fully characterize the tumor and could increase the risk of needle tract implantation metastases in the event of malignancy [52].

The therapy of oncocytic adrenocortical neoplasms mainly relies on surgical resection. With the development of laparoscopic techniques, laparoscopic surgery is becoming more and more popular [53]. It was suggested to perform the laparotomy when the tumor size is more than 6 cm to obtain a complete resection without tumor rupture [7].

Macroscopically, most of them were large, rounded, encapsulated, and well circumscribed tumors. On cut section, they were yellow brownish; some may have exhibited areas of hemorrhage or necrosis. The section of benign tumors was usually golden or brownish yellow, and the malignant tumors were mostly ash red or fish-meat-like. Microscopically, they were composed of large eosinophilic and granular cytoplasm cells with a central pyknotic nucleus, with a solid, trabecular, tubular, or papillary growth pattern.

The immunohistochemical pattern proved challenging to establish in many reported cases, as it had not been well-documented. Generally, these tumors are positive for vimentin, melan-A, synaptophysin, and alpha-inhibin, while they test negative for chromogranin.

Among the various molecular and cytological markers that could aid in the differential diagnosis of adrenocortical carcinoma, cell proliferation markers like Ki-67 and the oncoprotein p53 can be particularly valuable. Unfortunately, their role as definitive biomarkers remains uncertain due to the limited availability of these tumors [10].

However, as per Mearini et al., the Ki-67/MIB1 index is a practical tool for distinguishing between benign masses and carcinomas in the differential diagnosis [54].

The current study presented a limitation. Since eosinophilic cytoplasm may be caused by the prominence of a number of cytoplasmic organelles other than mitochondria (as occurs in oncocytic tumors), whether the oncocytic nature of the tumor cells comes from mitochondria should be confirmed. Therefore, nowadays, diagnosis also makes use of IHC with antimitochondrial antibody mES_13 in more than 90–95% of the tumor cells and/or electron microscopy. The definitive pathology in the current study (morphologically and immunohistochemically) was performed in a conventional way, and the diagnosis was reached by exclusion.

## 6. Conclusions

Adrenal oncocytic neoplasms are indeed rare tumors, with approximately 287 cases documented in the literature. They tend to be more prevalent in young women, often occurring in the fourth and fifth decades of life. Furthermore, they show a higher frequency in the left adrenal gland, and approximately 68% of cases are non-functional.

Regarding prognosis, oncocytoma (AO) and oncocytic neoplasm of uncertain malignant potential (AONUMP) generally have an excellent prognosis. In contrast, adrenal oncocytic carcinomas (AOCs) have a considerably higher risk of local relapse (5.1%), distant metastasis (3.7%), and mortality (7.4%). Effective management relies on a combination of biochemical, histopathologic, radiologic, and clinical features. However, surgical resection remains the primary treatment for adrenal oncocytic neoplasms. Surgical decisions should take into account the mass’s imaging characteristics, the patient’s age, and any concurrent medical conditions.

In general, the available data are of limited guidance in deciding when surgery is necessary. At diagnosis, in fact, oncocytomas are typically large, with a median size of 8.75 cm, possibly because they do not produce hormones and are difficult to distinguish from malignant adrenal tumors using imaging methods like CT or MRI. The usual density cutoff of <10 Hounsfield units (HU) to identify benign lesions on CT scans is inadequate for identifying benign adrenal oncocytomas, which have an average density of 14 HU. Additionally, the characteristic loss of signal on MRI out-of-phase images, often seen in benign adrenal lesions, is frequently absent in non-malignant oncocytomas. Consequently, most adrenal oncocytomas would be considered for surgery due to their indeterminate preoperative imaging characteristics. However, it is advisable to consider adrenalectomy if the adrenal mass reaches a diameter of 6 cm or more, if the mass increases by 1 cm or more during the observation period, or if there is evidence of autonomous hormonal secretion. During surgical procedures, the primary goal is to achieve complete resection of the adrenal gland without disrupting the adrenal capsule. Experienced pathologists play a crucial role in the pathological examination, allowing for the classification of tumors based on their biologic potential and providing insights into the patient’s prognosis.

## Figures and Tables

**Table 1:** Demographic data of patients with adrenal oncocytic neoplasms (AONs).

Characteristics	AONs (n = 287)	A/NA
**Gender**		282/5
*Females*	185 (65.6%)	
*Males*	97 (34.3%)	
**Age (years)**	44.2 (3.5–87)	285/2
**Location**		246/41
*Left*	143 (58.1%)	
*Right*	100 (40.6%)	
*Bilateral*	3 (1.2%)	
**Functionality**		272/15
*Non-functional*	185 (68%)	
*Functional*	87 (32%)	
**Size (mm** *)*	8.9 (2–28)	244/43
**Weight (g)**	467 (8–5720)	54/133
**LWB system**		278/9
*AO*	117 (42%)	
*AONUMP*	78 (28%)	
*AOC*	83 (30%)	

Abbreviation: A/NA, available/not available; LWB, Lin–Weiss–Bisceglia system; AO, oncocytoma; AONUMP, oncocytic neoplasm of uncertain malignant potential; AOC, oncocytic carcinoma.

**Table 2:** Functional adrenal oncocytic neoplasms (AONs): syndrome and secretion.

	Functional AONs (n = 87)	% (n = 272)
*Hyperandrogenism*	36	13.2
*Cushing syndrome*	22	8
*Hypercortisolism*	9	3.3
*Suppressed ACTH levels*	3	1.1
*Hypercortisolism + Hyperandrogenism*	8	2.9
*Mimicking Pheochromocytoma*	5	1.8
*Conn syndrome*	2	0.7
*IL6 secretion*	2	0.7

**Table 3:** Follow-up of all published AONs classified according to the LWB criteria.

Classification	n (%)	Median Follow-Up (m)	Range of Follow-Up (m)	Local Recurrence [n (%)]	Metastasis [n (%)]	Tumor-Related Deaths [n (%)]
**AO**	58 (43)	41.3	3–180	2 (1.5)	0	0
**AONUMP**	47 (35)	34.8	5–180	1 (0.7)	1 (0.7)	2 (4.2)
**AOC**	30 (22)	32.5	1–180	7 (5.1)	5 (3.7)	9 (30)
**Total**	135	37.1	1–180	10 (7.4)	6 (4.4)	11 (8.1)
Unclassifiable	152	NA	NA	NA	NA	NA

Abbreviations: NA, not available; AO, oncocytoma; AONUMP, oncocytic neoplasm of uncertain malignant potential; AOC, oncocytic carcinoma.

**Table 4:** Characteristics of death in the population with oncocytic adrenal neoplasms.

Authors	Age	Sex	Location	Sizec m	Weight g	Functional	LWB System		Tumor-Related Deaths (m)	Recurrence/Metastasis
Son SH (2013) [32]	53	m	left	9.8	NA	Cushing	AONUMP	Necrosis	15	Local and hepatic
Wong DD (2011) [22]	36	w	left	14	885	NO	AOC	NA	1	
	47	w	left	10	552	NO	AOC	NA	9	
	41	w	left	28	5720	Virilization	AOC	NA	16	Hepatic
	68	w	Right	8	70	NO	AOC	NA	21	Local
Duregon (2011) [33]	42	w	left	16	530	NO	AONUMP	Dimension + Necrosis	15	
	32	m	Right	23		NO	AOC	>5 mitotic figures per 50 HPFs atypical mitoses	24	
Bisceglia M (2004) [6]	46	m	Right	17	190	NO	AOC	>5 mitotic figures per 50 HPFs atypical mitoses	58	Local
	62	w	left	8	260	NO	AOC	>5 mitotic figures per 50 HPFs atypical mitoses	6	
Hoang MP (2002) [34]	53	w	left	17	1200	NO	AOC	Venous invasion	24	Bone
Seo IS (2002) [35]	49	w	left	8,5	200	MO	AOC	NA	72	Carcinosis
MEAN	48	8/3	8/3	14.5	1067	9/2	9/2		23.7	

Abbreviations: AONUMP, oncocytic neoplasm of uncertain malignant potential; AOC, oncocytic carcinoma; NA, not applicable due to availability of data.

## Data Availability

The datasets used and/or analyzed during the current study are available from the corresponding author on reasonable request.
